# Molecular Subtype-Specific Expression of MicroRNA-29c in Breast Cancer Is Associated with CpG Dinucleotide Methylation of the Promoter

**DOI:** 10.1371/journal.pone.0142224

**Published:** 2015-11-05

**Authors:** Elizabeth Poli, Jing Zhang, Chika Nwachukwu, Yonglan Zheng, Babatunde Adedokun, Olufunmilayo I. Olopade, Yoo-Jeong Han

**Affiliations:** Center for Clinical Cancer Genetics and Global Health, Section of Hematology/Oncology, Department of Medicine, University of Chicago, Chicago, IL, United States of America; University of Navarra, SPAIN

## Abstract

Basal-like breast cancer is a molecularly distinct subtype of breast cancer that is highly aggressive and has a poor prognosis. MicroRNA-29c (miR-29c) has been shown to be significantly down-regulated in basal-like breast tumors and to be involved in cell invasion and sensitivity to chemotherapy. However, little is known about the genetic and regulatory factors contributing to the altered expression of miR-29c in basal-like breast cancer. We here report that epigenetic modifications at the miR-29c promoter, rather than copy number variation of the gene, may drive the lower expression of miR-29c in basal-like breast cancer. Bisulfite sequencing of CpG sites in the miR-29c promoter region showed higher methylation in basal-like breast cancer cell lines compared to luminal subtype cells with a significant inverse correlation between expression and methylation of miR-29c. Analysis of primary breast tumors using The Cancer Genome Atlas (TCGA) dataset confirmed significantly higher levels of methylation of the promoter in basal-like breast tumors compared to all other subtypes. Furthermore, inhibition of CpG methylation with 5-aza-CdR increases miR-29c expression in basal-like breast cancer cells. Flourescent *In Situ* Hybridization (FISH) revealed chromosomal abnormalities at *miR-29c* loci in breast cancer cell lines, but with no correlation between copy number variation and expression of miR-29c. Our data demonstrated that dysregulation of miR-29c in basal-like breast cancer cells may be in part driven by methylation at CpG sites. Epigenetic control of the miR-29c promoter by epigenetic modifiers may provide a potential therapeutic target to overcome the aggressive behavior of these cancers.

## Introduction

Breast cancer affects more than 230,000 women, and results in nearly 40,000 deaths, making it the second deadliest cancer in women behind lung cancer [1]. Breast cancer is now known to be a group of heterogeneous diseases, consisting of neoplasms with vastly different molecular subtyping, gene expression profiling, clinical characteristics, prognosis, and response to various treatments [1,2,3,4,5,6]. With the advent of genome-wide studies, high-throughput sequencing, and microarrays, it is now known that the five molecular subtypes of breast cancer- luminal A, luminal B, basal-like, claudin-low, and HER2 enriched- represent diverse disease processes[2,5,6]. Recent studies have further stratified breast cancers into ten clusters based on the impact of somatic copy number aberrations (CNAs) on the transcriptome [7,8]. Basal-like tumors are defined by high expression of cytokeratins 5/6 and 17, low expression of the estrogen receptor (ER) and HER2 receptor, and aberrant global DNA hypermethylation [9,10] Clinically, basal-like breast cancer (BLBC) is associated with a more aggressive and metastatic behavior with a poorer prognosis and shorter survival time, and has a higher prevalence among premenopausal African American women [10,11]. Recent genome-wide studies have clustered basal-like tumors by the propensity to harbor *TP53* mutations and have a significant amount of DNA copy number variation [6]. An understanding of the genetic and molecular aberrancies unique to BLBC will help to optimize therapeutic regimens to increase survival in these patients.

MicroRNAs (miRNAs) are short (16–29 base pairs), non-protein coding RNA transcripts that play important roles in regulating genes involved in human development, health, and disease [12]. It has been predicted that miRNAs target 5,300 genes, which is nearly a third of the human gene set [13]. Reports in the last decade have revealed how the dysregulation of miRNAs can play a critical role in cancer initiation and progression [14,15]. Aberrant expression of miRNAs can serve as either oncogenes or tumor suppressors by disrupting cell proliferation, apoptosis, angiogenesis, the immune response, and the epithelial to mesenchymal transition [16].

MicroRNA-29c (miR-29c) is part of a microRNA family, which also includes mir-29a and mir-29b-1/2. This group of microRNAs has been extensively studied and has been shown to be involved in a vast range of diseases, including atrial fibrillation, hepatic fibrosis, ischemic brain injury, and endometriosis [17, 18, 19, 20]. MiR-29c has also been shown to be downregulated in several cancers, such as gastric, peripheral nerve sheath tumors, esophageal squamous cell carcinoma, melanoma, and breast cancer, among others [12, 21, 22, 23]. In these diseases, the decreased expression of miR-29c leads to the dysregulation of its downstream targets that are involved in epigenetic modification, metastasis, and cell proliferation, such as DNMT, B7-H3, RCC2, and cyclin E [12, 22, 24, 25, 26].

Research has shown that miR-29c is an integral regulator of several cellular pathways and its role in cancer is undoubtedly complex. We and others have shown that miR-29c is specifically downregulated in basal-like breast cancer, compared to other subtypes [12]. However, the etiology of this altered expression has not been explained. Studies have shown that the miR-29b-1/miR-29a promoter on Chromosome 7 is suppressed by c-Myc, Hedgehog, and NF-kappaB [27]. However, the miR-29b-2/miR-29c locus is on Chromosome 1 and its promoter has not been previously identified to determine the regulatory factors involved in its altered expression in cancer. In this study, we located the promoter of miR-29b-2/miR-29c to be 20kb upstream of the genes. Bisulfite sequencing of CpG sites in the promoter revealed hypermethylation of the miR-29c promoter in basal-like breast cancer cell lines and expression of miR-29c in basal-like cell lines responded to treatment with 5-aza-CdR. These results suggest that epigenetic changes may affect for the difference in expression of miR-29c in basal and luminal breast cancers, suggesting potential for regulation of miR-29c expression by epigenetic modifiers to be used as a therapeutic target for BLBC.

## Materials and Methods

### Cell culture and total RNA, miRNA, and DNA isolation

Human mammary epithelial (HMEC) primary cells were purchased from Lonza and breast cancer cells were obtained from the American Type Culture Collection (ATCC, Manassas, VA). All cell lines were tested negative for mycoplasma contamination and validated for species and unique DNA profile using short tandem repeat (STR) analysis by the provider or us. Breast cancer cell lines 184A1, HMEC, HCC1428, T47D, ZR7530, AU565, HCC202, HCC70, UACC3199, DU4475, HCC1937, HS578T, HCC38, and MDAMB231 were cultured and maintained in the specified media (S1 Table). When cells reached 60–70% confluence, microRNAs were extracted using the Qiagen miRNAeasy mini kit (Qiagen, Montgomery, MD). Total RNA and DNA were isolated from cell lines using the Qiagen RNA/DNA mini kit. The integrity of RNAs was validated by bio-analyzer at the University of Chicago Genomics Core Facility. RNAs with minimum RNA integrity number of 8 were applied to cDNA synthesis.

### qRT-PCR

cDNA synthesis from mRNA was performed using the SuperScript III First-Strand synthesis Super Mix (Invitrogen, Carlsbad, CA). Reverse transcription reactions for miRNAs were performed using the Taqman microRNA reverse transcription kit (Applied Biosystems, Foster City, CA). Taqman miRNA assays (Applied Biosystems) were used to detect mature form of miR-29c. After testing three different endogenous controls, we selected mir-103 as an internal control because its expression has minimal variation across cell lines. All real-time PCR were performed in triplicate, and the fold change in expression of miRNAs was calculated using the ΔΔCt method, with miR-103 as an endogenous control.

### Identification and molecular cloning of the promoter

The transcription start site (TSS) for miR-29c/29b-2 was identified by the publicly available Cap-Analysis Gene Expression (CAGE) database (http://fantom3.gsc.riken.jp). The UCSC genome browser was also utilized to analyze histone modification profiles in the promoter/enhancer region (http://genome.ucsc.edu). On the basis of the analyses, the promoter region of miR-29c/b-2 was amplified by PCR using a BAC clone (CTD2379P21) as a template with 2862F (5’-GTGCCGAAAGGAAGAC-3’) and 3486R (5’-TCTTTAGGGGTGTGCGTAGG-3’) primers. Respective amplicons were then cloned into a pGL3-Basic firefly luciferase vector at MluI and Xho I sites.

### Reporter activity assays and chromatin immunoprecipitation (ChIP) assays

For dual luciferase reporter gene assays, cells grown in 6-well plates were cotransfected with 2 μg of the firefly luciferase vector containing miR-29c promoter and 10 ng of CMV-renilla luciferase vector (Promega, Madison, WI, USA) using Dharmafect Duo transfection reagent (Dharmacon, Lafayette, CO). For chromatin immunoprecipitation (ChIP) assays, cells were cross-linked, sonicated, and immunoprecipitated with an anti-RNA Polymerase II antibody using Magna ChIP kit (Millipore, Billerica, MA, USA) according to the manufacturer’s protocols. Normal mouse IgG was used as a negative control.

### Bisulfite sequencing

CpG sites in the miR-29c promoter were determined from ENCODE data available on the UCSC genome browser, and MethPrimer software [28]. Primers were designed that flanked these region (S2 Table). Bisulfite modification of 1 μg of DNA from 13 selected breast cancer cell lines was carried out with the EZ Methylation Gold™ kit from Zymo Research (Irvine, CA) according to the manufacturer’s protocol. The PCR products were run on a gel to confirm specificity. After cleaning PCR products with Exonuclease I (New England BioLabs Inc, Ipswich, MA) and Shrimp Alkaline Phosphatase (Affymetrix, Santa Clara, CA), Sanger sequencing was performed at the University of Chicago Comprehensive Cancer Center DNA Sequencing and Genotyping Facility. Chromatograms of DNA sequencing were read on Chromas Lite 2.1.1 (Technelysium, South Brisbane, Australia). Substituted thymines for all cytosines at non-CpG sites confirmed successfulness of bisulfite conversion. When sequencing results for a few of the cell lines did not result in clear chromatograms, the PCR amplicons from these samples were cloned into a pCR2.1-TOPO vector and four cloned colonies were chosen for sequencing. The number of methylated CpG sites was averaged for these cell lines.

### Treatment with 5-Aza-2’-Deoxycytidine

Basal-like breast cancer cell lines (UACC3199, DU4475, HCC70, and HCC1937) and the luminal cell line (AU565), were treated in triplicate for five days with 5-Aza-2’-Deoxycytidine (5-aza-CdR) at a 100 nM dose in water. Control samples were treated with water. On the sixth day, RNA was extracted for qRT-PCR analysis.

### The Cancer Genome Atlas (TCGA) data and statistical analysis

TCGA breast invasive carcinoma copy number variant (CNV, GISTIC2 method, version 2013-06-02), RNA sequencing (IlluminaHiSeq miRNASeq level 3.1.16.0, version 2014-05-26), and DNA methylation (Illumina Infinium HumanMethylation450 BeadChip, version 2014-05-02) datasets were extracted from UCSC Cancer Browser (https://genome-cancer.ucsc.edu/), along with the clinical-pathological phenotypes. Out of 838 patient samples with DNA methylation profile, only 588 samples (70 normal breast tissues, 124 luminal B, 276 luminal A, 31 HER-2 and 87 basal-like breast tumors) were included in the analysis, after excluding samples from male patients, samples with no PAM50 subtype information, or samples showing discrepancy between PAM 50 subtypes and pathological analysis.

We performed the one way ANOVA and Kruskal-Wallis testes to compare the methylation level across the breast tumor subtype groups. Tamhane's post hoc test was performed for multiple comparisons between two subtype groups. Statistical significance was set at P < 0.05. Statistical analysis was carried out by SPSS for Windows ver.16 (SPSS Inc., Chicago, IL), and plots were generated by Graphpad Prism 6.0 (GraphPad Software, Inc., La Jolla, CA).

### Fluorescent *In Situ* Hybridization (FISH) with home-brewed probes

To evaluate miR-29c copy number in breast cancer cell lines, we carried out FISH using a home-brewed probe containing the BAC CTD2379P21 (Life Technologies, Carlsbad, CA). The probe was prepared using the Abbott Nick Translation Kit (Abbott Laboratories, Abbott Park, IL) and labeled with Spectrum Green. The *miR-29c* probe was mixed with *CEP1* (centromere for chromosome 1) in Spectrum Orange (centromere enumeration probe for Chromosome 1, Vysis/Abbott Laboratories). Breast cancer cell lines were arrested in metaphase and harvested according to standard protocols [29]. Chromosomes were identified by 4',6-diamidino-2-phenylindole (DAPI) staining. Chromosomal mapping and hybridization efficiency for the probe mixture was verified in metaphase spreads and interphase nuclei of a normal lymphoblastoid cell line. Cell lines were scored and digital images were obtained using the Zeiss AXIO IMAGER Z2 microscope and Zeiss AxioCam MRm Rev 3 Monochromatic Camera. The images were merged and colored using Adobe Photoshop software (Adobe Systems, San Jose, CA). For each cell line, signals were counted in 60 metaphase and interphase cells with well-defined nuclei and averaged. The ratio of the *miR-29c* signal to *CEP1* signal and percentage of each signal pattern was used to determine copy number in each cell line. Polysomy was defined as greater than three copies of a chromosome. The miR-29c:CEP1 ratio ranges for deletion and amplification are as follows: deletion is < 0.8, >1 and < 2 is gain, and >2 is amplification.

## Results

### MiR-29c is down-regulated in basal-like breast cancer

**Fig 1:**
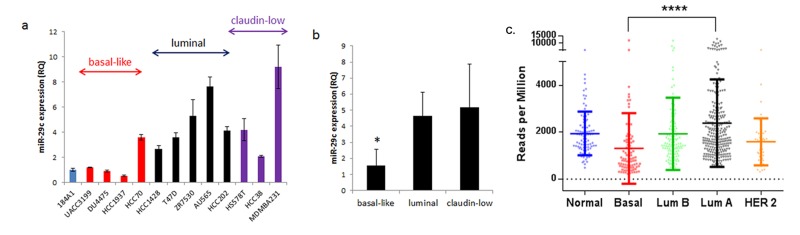
Expression of miR-29c in breast cancer cells. (a) qRT-PCR results of miR-29c expression in breast cancer cell lines relative to expression in normal breast epithelial cell line 184A1 (RQ, relative quantity). MiR-29c expression organized by subtype reveals decreased expression in basal-like breast cancer cell lines (red), compared to luminal cell lines (black) and claudin-low cell lines (purple). (b) Expression of miR-29c in basal-like cell lines is statistically significantly lower than expression in luminal and claudin-low (p = .015). (c) TCGA data showed significantly lower expression of miR-29c in basal-like tumors compared to luminal A tumors (****p< 0.0001).

To characterize expression of miR-29c in breast cancer subtypes and verify previous studies that have shown that miR-29c is expressed lower in the basal-like breast cancer subtype [12], we examined expression of the microRNA in breast cancer cell lines using qRT-PCR. Expression was measured in 5 luminal (HCC1428, T47D, ZR7530, AU565, and HCC202), 4 basal-like (HCC70, UACC3199, DU4475, and HCC1937), and 3 claudin-low cell lines (HS578T, HCC38, MDAMB231), relative to expression of miR-29c in 184A1, which is an immortalized breast epithelium cell line (Fig 1A). In aggregate, expression of miR-29c was found to be significantly lower in basal-like cell lines compared to luminal and claudin-low (Fig 1B). To validate our data in a larger sample set, we utilized primary breast tumor samples from TCGA network research consortium [7]. Analysis of *miR-29c* expression in breast tumors using TCGA Illumina HiSeq dataset confirmed lower expression of miR-29c in basal-like tumors in comparison to luminal A tumors (Fig 1C).

### The miR-29c promoter is located 20kb upstream from miR29b-2/29c gene

**Fig 2:**
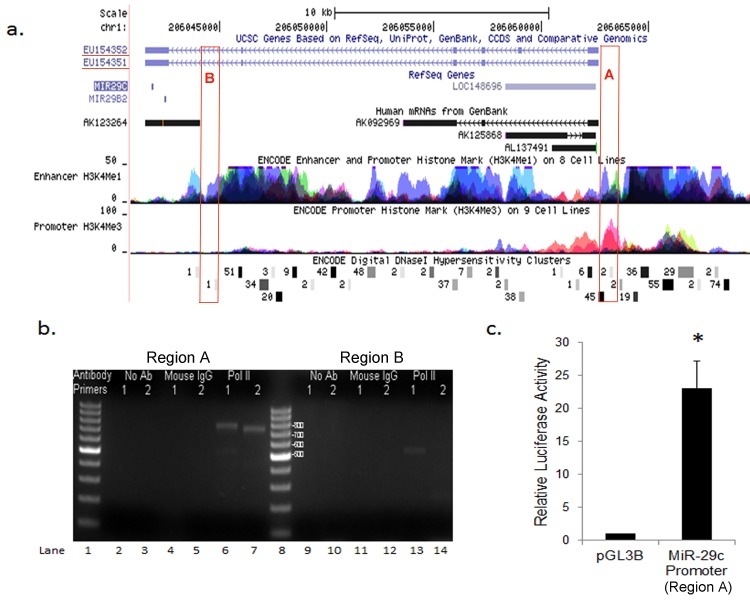
Analyses and Identification of MiR-29c Promoter. (a). UCSC genome browser shows location of the miR-29c/29b2 gene at chromosome 1q32. Two primary transcripts are shown with GenBank ID, EU154352 and EU154352. Potential promoter region located 20 kb (region A) or 1Kb (region B) upstream of the gene with 6 or 1 CAGE tags, respectively, were indicated with red boxes. The enhancer (H3K4me1) and promoter (HeK4Me3) markers of histone modifications from ENCODE are also shown. (b). ChIP against RNA Polymerase II (Pol II) revealed enrichment of Pol II at the 20kb-upstream promoter (region A). PCR were performed using two primer sets (primer set 1 and 2) targeting at each promoter. ChIP without any antibody (No Ab) or with an antibody against Mouse IgG was used as a negative control. (c) Luciferase activity was measured in MDA-MB-231 cells transfected with pGL_3_-Basic luciferase reporter vectors or miR-29c promoter-luciferase constructs. The means±SD from three experiments are shown.

To determine if epigenetic modifications at the miR-29c promoter drives the lower expression of miR-29c in basal-like breast cancer, we first identified and characterized the miR-29c promoter in breast cancer cells. Using CAGE tag data, which is based on preparation and sequencing of concatemers of DNA tags derived from the initial 20 nucleotides from the 5′ end of mRNA, we were able to identify two areas of upstream of the *miR-29b-2/29c* gene that were potential TSS of miR-29c/29b2 (Fig 2A). Region A (~20 kb upstream of miR-29c) had six CAGE tags, while region B (~1kb upstream) had one CAGE tag. When we analyzed chromatin signatures of transcriptional promoters and enhancers [30, 31] from ENCODE dataset, we found the presence of a marker of histone modification (H3K4me1) in the regions both regions A and B. In contrast, an active promoter marker of histone modification (H3K4me3) was only identified in region A (Fig 2A). To validate *in silico* analysis experimentally, we performed ChIP assays using antibodies against RNA polymerase II and mouse IgG, followed by PCR with two independent primer sets targeting at each region. Enrichment of RNA polymerase II was observed on the region A (lanes 6 & 7, Fig 2B) with no enrichment in the absence of antibody (lanes 2–3, Fig 2B) or in the presence of mouse IgG controls (lanes 4–5, Fig 2B). Region B showed weak enrichment of RNA polymerase II only with primer set 1(lane 13, Fig 2B).

Two primary transcripts possibly originating at the 20kb-upstream promoter Region A were reported in GenBank (EU154352 and EU154352), which were described as Homo sapiens microRNA 29b2/29c precursor RNA. According to the UCSC genome browser (hg18), the cDNA of EU154352 (FLJ35650 fis, clone SPLEN20136440) is 21,182 bp from 206,041,490 to 206,062,671 at Chromosome 1.

We next cloned the promoter region A into pGL_3_-basic reporter vector and assessed the promoter activities in MDA-MB-231 cells (Fig 2C). Reporter activity assays showed that the proximal region (−607 to TSS) exhibited strong promoter activity in luciferase activities compared to vector control. On the bases of *in silico* analyses, ChIP assays, transcript analyses and reporter gene assays, we concluded that miR-29c/29b2 has a promoter in region A, ~20 kb upstream of the gene.

### Bisulfite sequencing of mir-29c promoter reveals subset-specific methylation changes

**Fig 3:**
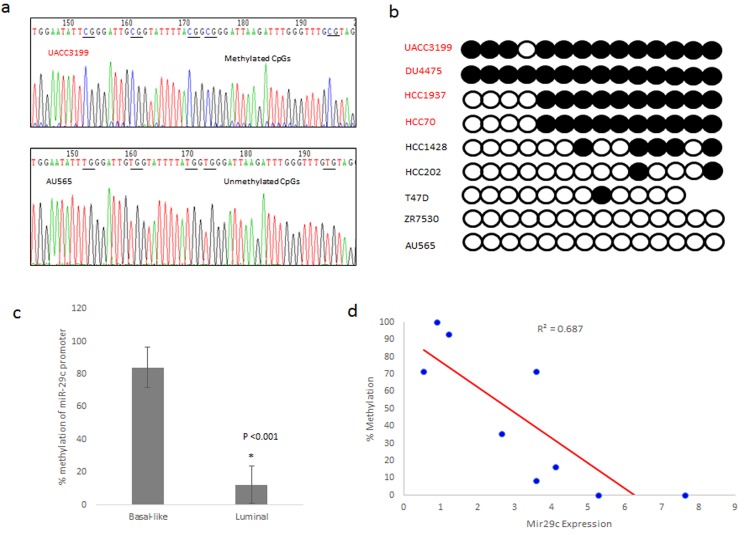
Methylation of the miR-29c promoter correlates with expression in basal-like cell lines. (a) Representative chromatographs of 50 base pairs in the miR-29c promoter after bisulfite treatment. The basal cell line UACC3199 contains cytosines in the sequence because methylation prohibited the effect of bisulfite treatment. AU565 is a luminal cell lines that has unmethylated cytosines in the miR-29c promoter, which results in the conversion of cytosine to thiamine after treatment with bisulfite. (b) Representative bisulfite sequencing analysis for the miR-29c promoter in breast cancer cell lines. Each circle represents a CpG dinucleotide in the promoter region. Methylated CpGs are represented by closed circles, while unmethylated CpGs are represented by open circles. (c) The percent of methylation of the sequenced regions of the miR-29c were averaged by subtype. The basal-like cell lines had a significantly higher percentage of methylation compared to luminal cell lines (p = 0.006). (d) There is an inverse correlation between expression of miR-29c and the percent of methylation of CpG dinucelotides in the promoter. Basal-like cell lines have higher methylation and lower expression, while luminal cell line had less methylation of the promoter and higher expression.

To discover whether specific epigenetic modifications of the miR-29c promoter result in dysregulation of miR-29c expression, potential CpG sites were located upstream of the transcription start site. A 309 base pair region with 14 CpG sites was amplified and subjected to bisulfite sequencing. A representative chromatograph of a basal-like (UACC3199) and a luminal cell line (AU565) show differences in sequence reading after bisulfite treatment (Fig 3A). Five CpG sites are all methylated in UACC3199 cells whereas, in AU565 cells, they are all unmethylated and converted into ‘TG’ with bisulfite treatment.

Analysis of methylation of 14 CpG sites showed that the basal-like cell lines (UACC3199, DU4475, and HCC1937) had 93%, 100%, and 71.4% methylation of the miR-29c promoter (Fig 3B). In contrast, the luminal cell lines (HCC1428, T47D, ZR7530, AU565, and HCC202) had methylation of 0% to 35.7% of the miR-29c promoter in the CpG sites sequenced. In total, the luminal cell lines had on average 12.1% methylation and basal-like cell lines had 83.9% methylation of the miR-29c promoter, showing statically significant difference between luminal and basal-like cells (p = .001) (Fig 3C). A representative chromatograph of each cell line is also shown in S1 Fig.

Correlation analysis in the breast cancer cell lines showed a significant inverse correlation between the percent methylation and the expression of miR-29c (Fig 3D). The basal-like cell lines with the lowest expression had a higher percentage of methylation, while luminal cell lines with higher expression of the miRNA had less methylation. These results indicate that promoter methylation of miR-29c may be a potential epigenetic modification resulting in expression differences seen between the basal-like and luminal subtypes of breast cancer.

### 5-Aza-2’-Deoxycytidine treatment of basal-like breast cancer cell lines increased expression of miR-29c

**Fig 4:**
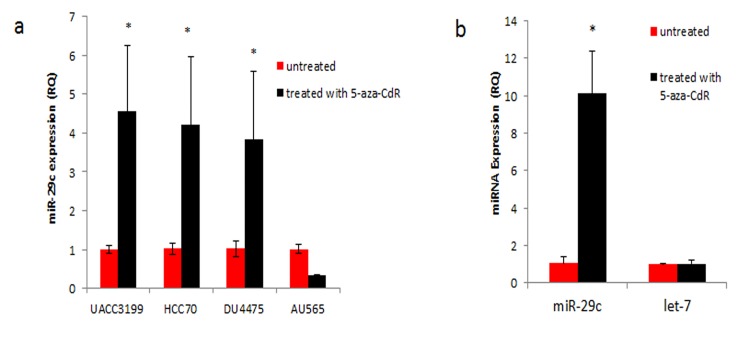
Treatment of breast cancer cell lines with 5-aza-CdR, results in increased expression of miR-29c in basal-like cell lines. (a) qRT-PCR results after cell lines were treated for five days with 5-aza-CdR. Basal-like cell lines with methylation of the miR-29c promoter had statistically significant higher expression of miR-29c after treatment. Expression of miR-29c was not increased in AU565, a luminal cell line with low methylation of the promoter. (b) There was no statistically significant difference in expression of microRNA let-7, which is not known to have promoter methylation, after treatment of 5-aza-CdR in UACC3199, a basal-like cell line.

To determine if de-methylation of the promoter could increase expression of miR-29c, we treated three basal-like breast cancer cell lines (UACC3199, DU4475, and HCC70) and a luminal cell line (AU565) with 5-aza-CdR. All treated basal-like breast cancer cell lines showed an increase in expression of miR-29c after treatment with the demethylation agent (Fig 4A). Of note, HCC1937 was also tested with 5-aza-CdR, but we were unable to obtain consistent results with this cell-line. We then tested if the treatment of 5-aza-CdR causes a change in the expression of miR-29c in AU565, which is already completely unmethylated in the miR-29c promoter. We found that there was no increase in expression of miR-29c in this cell line, confirming that this drug has no effect on expression of miR-29c in cell lines that are already unmethylated. To determine if effect of 5-aza-CdR was specific for methylated micro-RNAs, the expression of let-7 was measured in breast cancer cell lines before and after treatment. The results in Fig 4B show that there was no statistically significant difference in expression of let-7 after treatment of a basal-like cell line with the drug. Together, these results show that CpG methylation of the miR-29c promoter plays an important role in gene expression in breast cancer cell lines.

### TCGA confirms hypermethylation of the miR-29c promoter in human basal-like breast tumors

**Fig 5:**
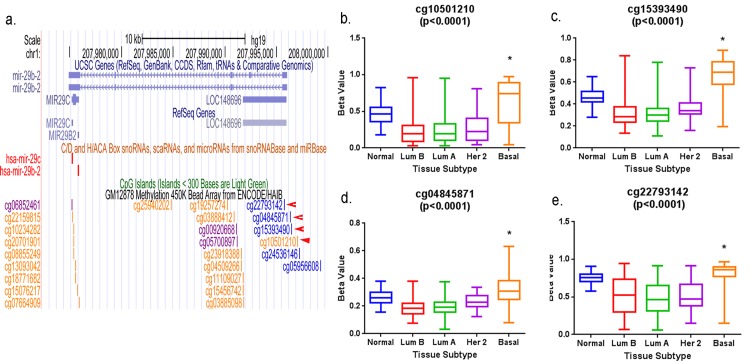
MiR-29c promoter is hypermethylated in human basal-like breast tumors. Methylation levels of four CpG dinucleotides in the promoter region of miR-29c were analyzed using the TCGA HumanMethylation450 Array data from 588 human breast tissues. These results are expressed as beta values, which are continuous variables between 0 and 1. Hypomethylated regions have lower beta values while hypermethylated regions have higher, more positive beta values. *Tamhane's post hoc and Tukey’s multiple comparison testes showed a significant difference in methylation levels of the CpG sites between basal-like tumors and other subtypes of breast tumors (p<0.05).

TCGA has published results of methylation analysis of human breast tumors using the Illumina Infinium HumanMethylation450 platform [7]. This analysis has probes for four CpG’s in the promoter region of miR-29c that match with four potentially methylated CpG dinucleotides published by ENCODE (Fig 5A). A one-way Anova and a Kruskal Wallis of the beta values at four of these probes (cg10501210, cg15393490, cg04845871, and cg22793142) showed a significant difference in methylation across four subtype groups (Fig 5B–5E). Tamhane's post hoc test showed significantly more methylation in the CpG sites of the promoters of basal-like breast tumors than luminal B, luminal A and HER-2 breast tumors. This data suggests that epigenetic regulation of the miR-29c promoter through methylation of CpG sites in that region can result in suppression of the expression of miR-29c in basal-like breast tumors.

### Copy number of miR-29c in breast cancer cell lines does not correlate with its expression

**Fig 6:**
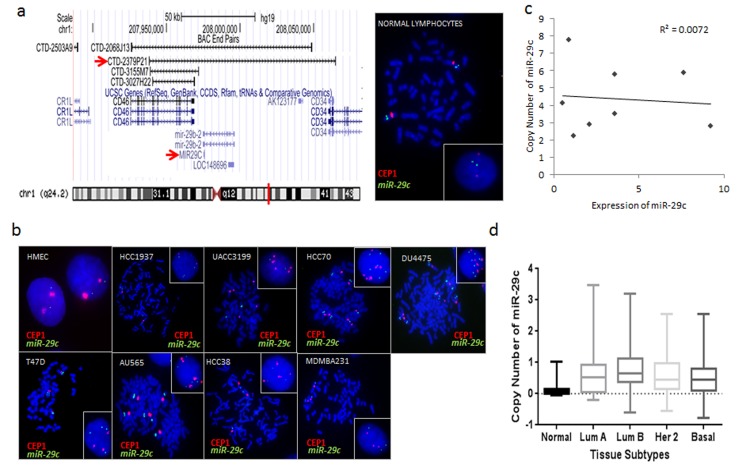
Fluorescent *In Situ* Hybridization (FISH) of miR-29c in breast cancer cell lines. (a) Genomic position of BAC CTD-2379P21 at chromosome 1q32. This clone was selected for a homebrewed miR-29c FISH probe. Right image shows photomicrograph of *miR-29c*:*CEP 1* FISH probes in control normal lymphocytes in metaphase and interphase (insert). (b) Photomicrographs of *miR-29c*:CEP1 in breast cancer cell lines are presented and show marked karyotype abnormalities. Cells were counterstained with DAPI (blue), while *miR-29c* is localized by green fluorescent signal, and *CEP1* is localized by a red flourescent signal. Metaphase and interphase (insert) cells are shown. These Results are summarized in Table 1 and described further in S1 Text. (c) Scatter plot showing no correlation between copy number of *miR-29c* (from FISH) and expression of *miR-29c* in the breast cancer cell lines. (d) TCGA copy number data analysis confirms no significant difference in copy number of *miR-29c* between the different breast cancer subtypes in human tumors.

Because basal-like tumors are known to have a significant copy number variation, we tested if subtype specific expression of miR-29c could be due to deletions or amplifications of the gene. FISH was performed using a home-brewed probe of BAC CTD2379P21, which contains *miR-29c* on 1q32 (Fig 6A). To test the probe hybridization efficiency, FISH was performed on the normal lymphocyte cell line GM14667. The mean *miR-29c* signal per cell was 1.95, and the *mir-29c/CEP1* ratio was 1.02 in the cell line with ninety percent of cells showing a normal pattern of two copies of each signal.

The normal breast epithelium cell line HMEC also showed a normal pattern of 2:2 (*miR29c*:*CEP1*, seen in 71.7% of cells) (Fig 6B). Although breast cancer cell lines displayed some degree of abnormal signal patterns including polysomy, gene rearrangement, or gene translocation, we detected no actual gene duplication or deletion of miR-29c in breast cancer cell lines. The results of our analysis are listed in Table 1 and described in detail in S1 Text. When we analyzed a correlation between copy number and expression of miR-29c, we failed to find any correlation in breast cancer cells (Fig 6C). Analysis of *miR-29c* copy number in breast tumors using TCGA GISTIC2 CNV dataset showed no significant difference in copy number of *miR-29c* among breast tumor subtypes (Fig 6D). Together, our data showed that *miR-29c* was not amplified in cell lines with higher expression, nor was it deleted in basal-like cell lines with lower expression, showing no correlation between copy number and expression of miR-29c.

**Table 1:** FISH results.

Cell line	Molecular subtype	Mir-29c/cell*	CEP1/cell	Ratio Mir-29c/CEP1	major representative cell clone (MiR29c:CEP1, %)	other representative clones (MiR29c:CEP1, %)	MiR29c amplification status	Interpretation
GM146	normal	1.9	1.9	1	2:2, 90%	1:1, 6.6%	No ampl	normal
HMEC	normal	1.9	2.1	0.9	2:2, 72%	1:2, 17%	No ampl	normal
UACC3199	basal	2.2	5	0.4	2:5, 58%	2:4, 13%	No ampl	gene translocation, CEP1 polysomy
HCC38	claudin-low	2.9	3.6	0.8	3:4, 48%	3:3, 20%	No ampl, gain	low polysomy
DU4475	basal	7.8	6.7	1.2	8:7, 42%	7:7, 17%	No ampl, gain	unbalanced and balanced polysomy
T47D	luminal	3.5	3	1.2	4:3, 50%	3:3, 38%	No ampl, gain	low polysomy, gene gain and rearrangement
HCC1937	basal	4.1	2.1	1.9	4:2, 72%	4:3, 10%	Equivocal, gain	gain
HCC70	basal	5.8	6.4	0.9	6:7, 50%	6:6, 27%	No ampl, gain	unbalanced and balanced polysomy, gene transolcation, CEP1 dicentric chromosome
AU565	luminal	5.9	5.7	1	6:6, 37%	6:5, 21.7%	No ampl, gain	balanced and unbalanced polysomy, CEP1 dicentric chromosome
MDAMB-231	claudin-low	2.8	2.9	0.9	3:3, 57%	2:3, 13.3%	no ampl, gain	low balanced polysomy

*60 cells were scored for each cell line.

## Discussion

In this study, we have confirmed that miR-29c is downregulated in basal-like breast cancer cell lines compared to luminal and claudin-low. We have also identified the promoter of *miR-29b-2/29c* to be 20kb upstream of the gene on Chromosome 1. Bisulfite sequencing of CpG sites in the miR-29c promoter revealed hypermethylation in basal-like cell lines compared to luminal cell lines, which correlated with expression difference. Basal-like cell lines treated with a hypomethylating agent, 5-aza-CdR, had higher expression of miR-29c compared to untreated samples.

DNA methylation is due to the covalent addition of methyl groups to cytosines in cytosine-guanine dinucleotides by DNA methyltransferases (DNMTs), which results in decreased expression of the gene, and is central to normal genetic regulation and cellular function [32]. However, in cancer, regulation of the methylation of gene promoters can be disturbed, resulting in global hypo or hypermethylation, as well as epigenetic changes specific to certain genes. It has been discovered that aberrant expression of microRNAs in cancer is often the result of changes in promoter DNA methylation of microRNA genes [33]. For example Baer *et al* discovered five microRNAs, miR-124-2, miR-129-2, miR-9-2, miR-551b, and miR-708, to be hypermethylated in chronic lymphocytic leukemia [34]. In addition, miR-10a has been found to be hypermethlyated in gastric cancer, and miR-203 is downregulated due to hypermethylation in endometrial cancer [35,36].

Human breast cancer cell lines are useful models for research because they can be used to model the molecular portraits of human breast cancer. However, breast cancer cell lines exhibit a large degree of heterogeneity in expression profiles and copy number variations, much like the tumors they mirror. While we found a statistically significant difference in miR-29c expression across subtypes, we also observed high variability of miR-29c expression across cell lines within each subtype. For instance, HCC70, a basal-like cell line, has higher expression than the other basal-like cell lines (Fig 1A). The data indicates heterogeneity within basal-like subtype, perhaps because of genetic and molecular variability of each cell line. In support of this, Pietenpol group identified six TNBC subtypes (TNBCtyping) through molecular profiling of TNBC tumors [37]. Nonetheless, when compared to other subtypes, our data clearly shows a statistically significant decreased expression of miR-29c in basal-like cell lines. Moreover, our cell-line data is confirmed by expression patterns of miR-29c in breast tumors using the TCGA database.

Expression of microRNA genes could be regulated genetically and epigenetically. After examining a genetic (copy number) and an epigenetic factor (CpG methylation) for miR-29c expression, we suggested that dysregulation of miR-29c in basal-like breast cancer cells may be in part driven by methylation at CpG sites. It is of note that HCC70 has the same methylation pattern as HCC1937 (Fig 3B) but higher expression of miR-29c compared to HCC1937 (Fig 1B). The data suggest that there are likely several different modifiers of mir-29c’s expression in these cell lines, including transcription factors potentially involved in regulation of the promoter activity. Nevertheless, our cell-line data is corroborated by methylation patterns of the miR-29c promotor in the TCGA database: breast tumors of the basal-like subtype were more likely to have methylation at CpG islands within the promotor region compared to luminal subtype tumors.

Despite the limitations stated above, this study is the first to discover the hypermethylation as a potential factor in the downregulation of miR-29c in basal-like breast cancer. Of note, miR-29c is itself a post-transcriptional regulator of DNMT3b, and others have shown that DNMT3b is overexpressed in breast cancer cell lines with a hypermethylator phenotype [12, 38]. Our results add to the complexity of this relationship, as methylation of the promoter of *miR-29c* in basal-like breast cancer can decrease miR-29c expression, and, therefore, cause deregulation and over-expression of DNMT3b. This can then result in hypermethylation of the genome.

The expression of miR-29c is implicated in several different disease processes and cancers, and it is often downregulated in these diseases. It is not known yet if the promoter of miR-29c in hypermethylated in these diseases as well. However, we have shown that the expression of miR-29c is increased in basal-like breast cancer cell lines with the treatment of 5-aza-CdR, which raises the question of the use of epigenetic modifying therapies in breast cancer. Currently, 5-aza-CdR and other drugs in this class, are frequently used in hematologic malignancies, and are being used in clinical trials for some solid tumors [39, 40]. More research is required to determine if the regulation of the expression of miR-29c by epigenetic modifiers will lead to a successful therapy in basal-like breast cancer.

## Supporting Information

S1 Fig Chromatographs of Bisulfite sequencing. (DOCX)

S1 Table Cell lines and cell culture conditions used. (DOCX)

S2 Table Bisulfite Sequencing Primers. (DOCX)

S1 Text Analysis of breast cancer cell line FISH. (DOCX)
