# Supplementary figures and images for: Molecular Subtype-Specific Expression of MicroRNA-29c in Breast Cancer Is Associated with CpG Dinucleotide Methylation of the Promoter
Source: PLoS One. 2015 Nov 5;10(11):e0142224. doi: 10.1371/journal.pone.0142224 (PMC4634951; doi:10.1371/journal.pone.0142224)

**S1 Figure. Chromatographs of Bisulfite sequencing**

**
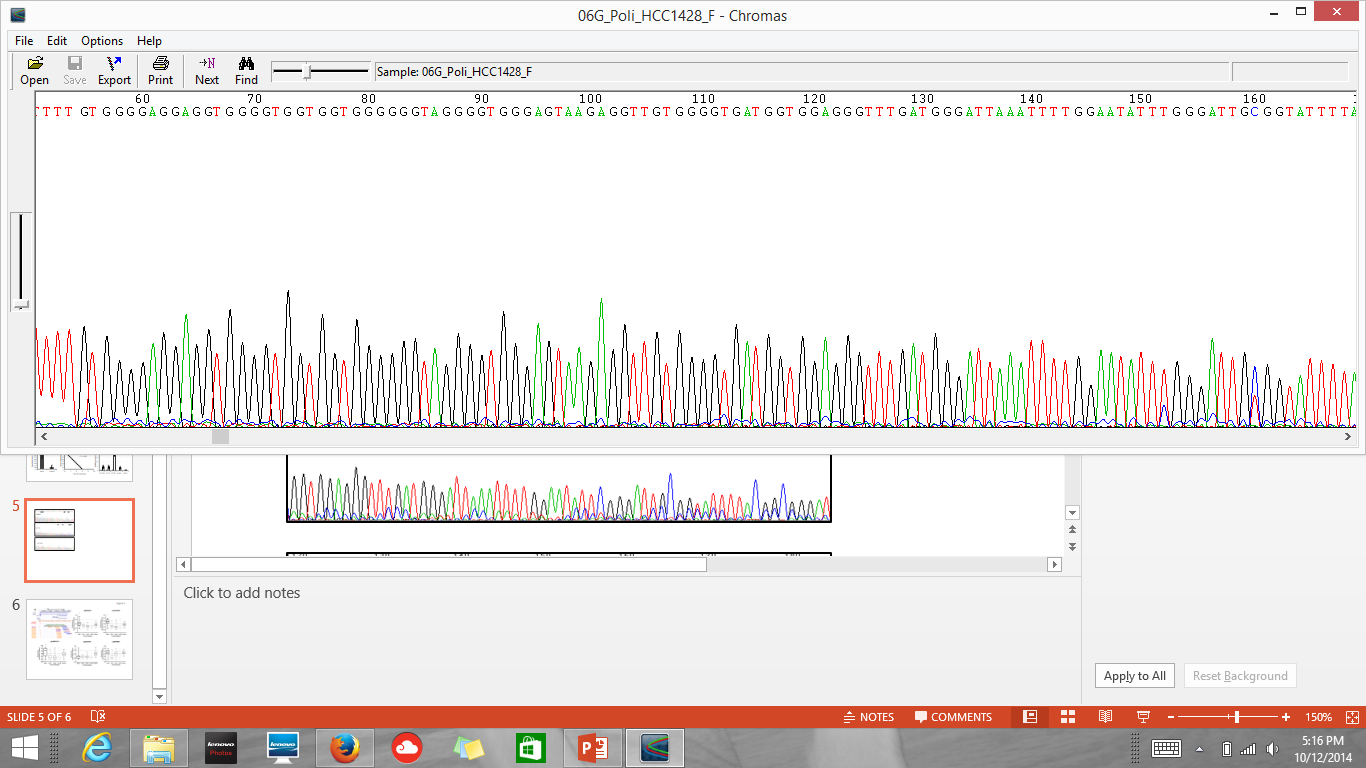
**
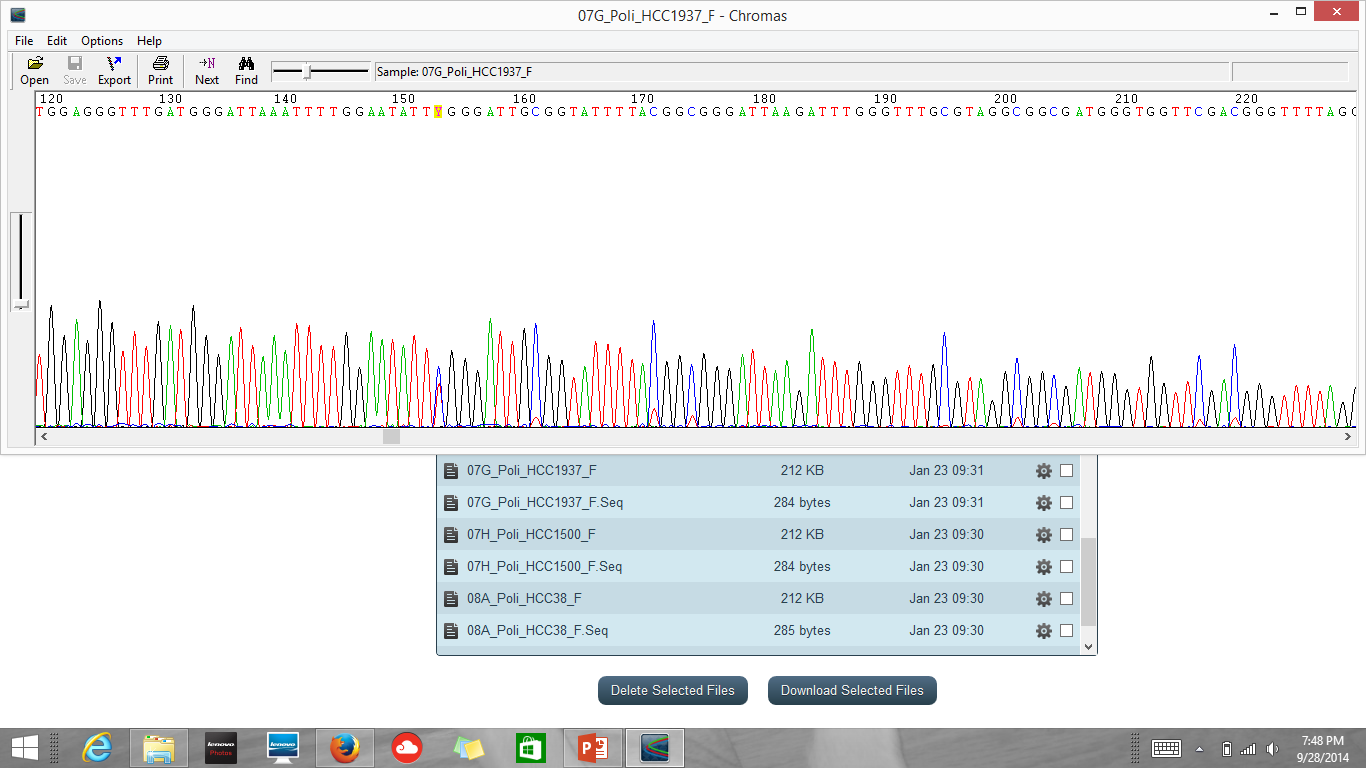

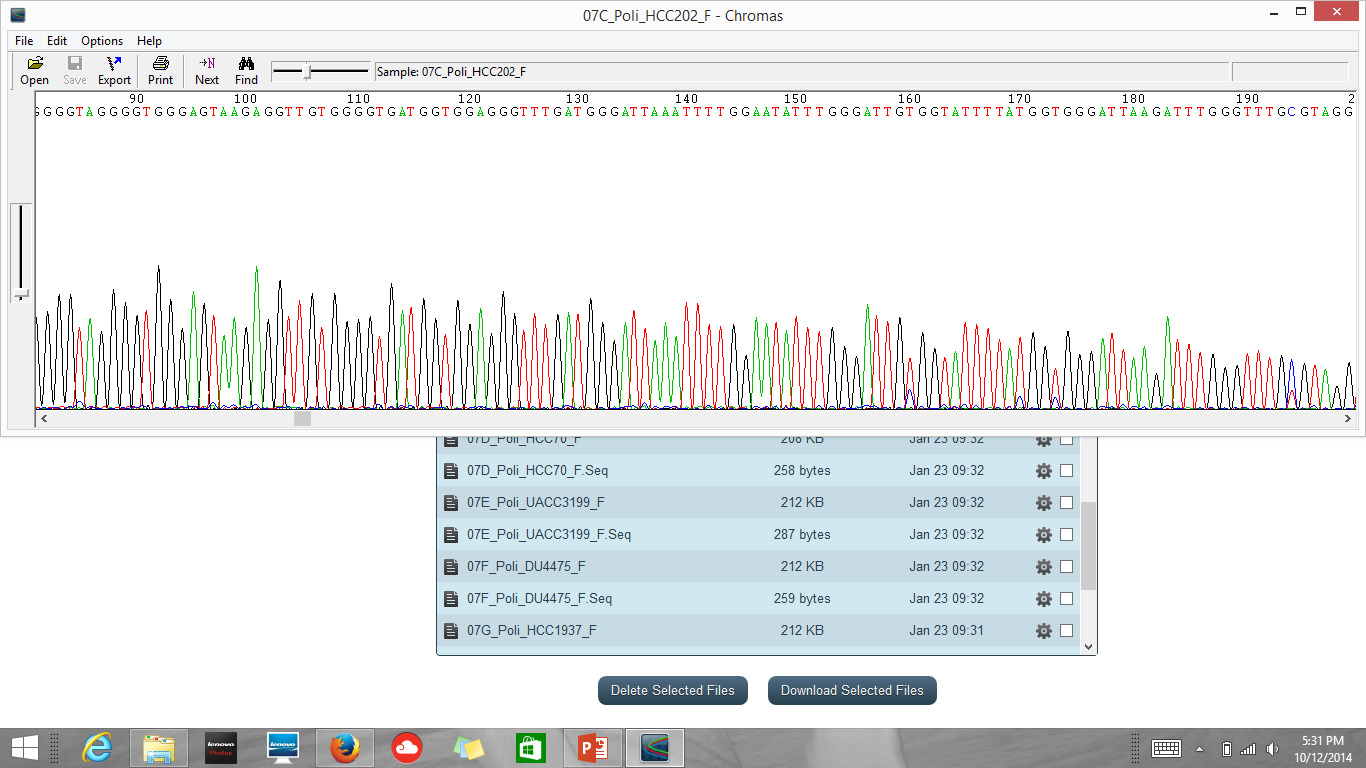

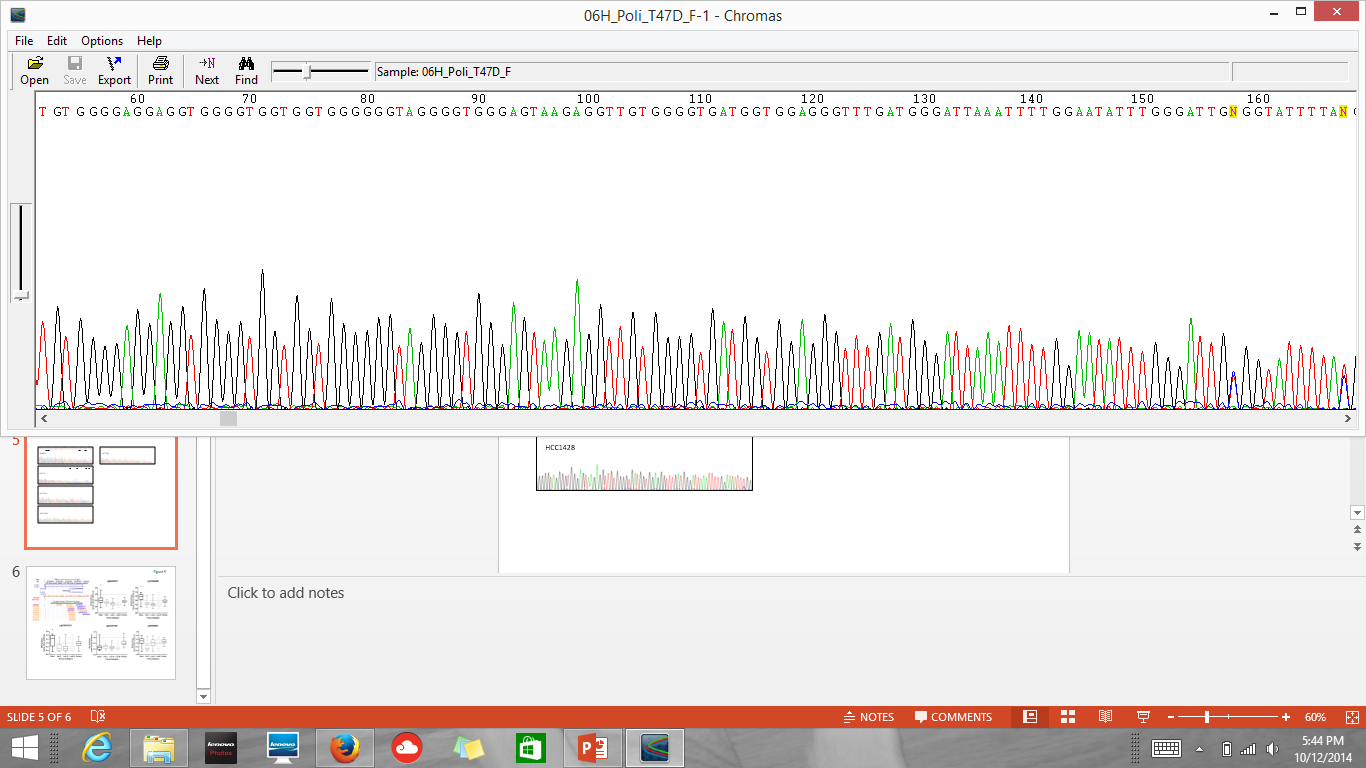

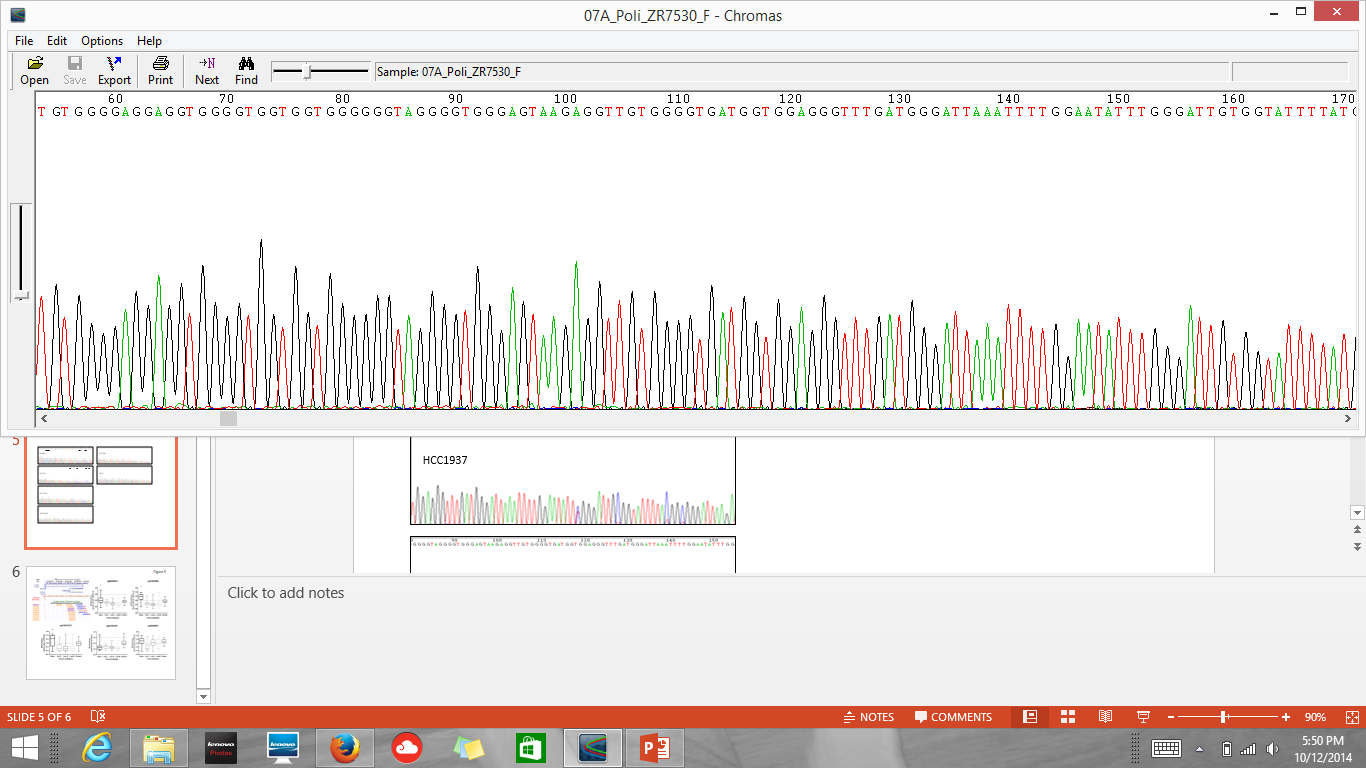


HCC1428


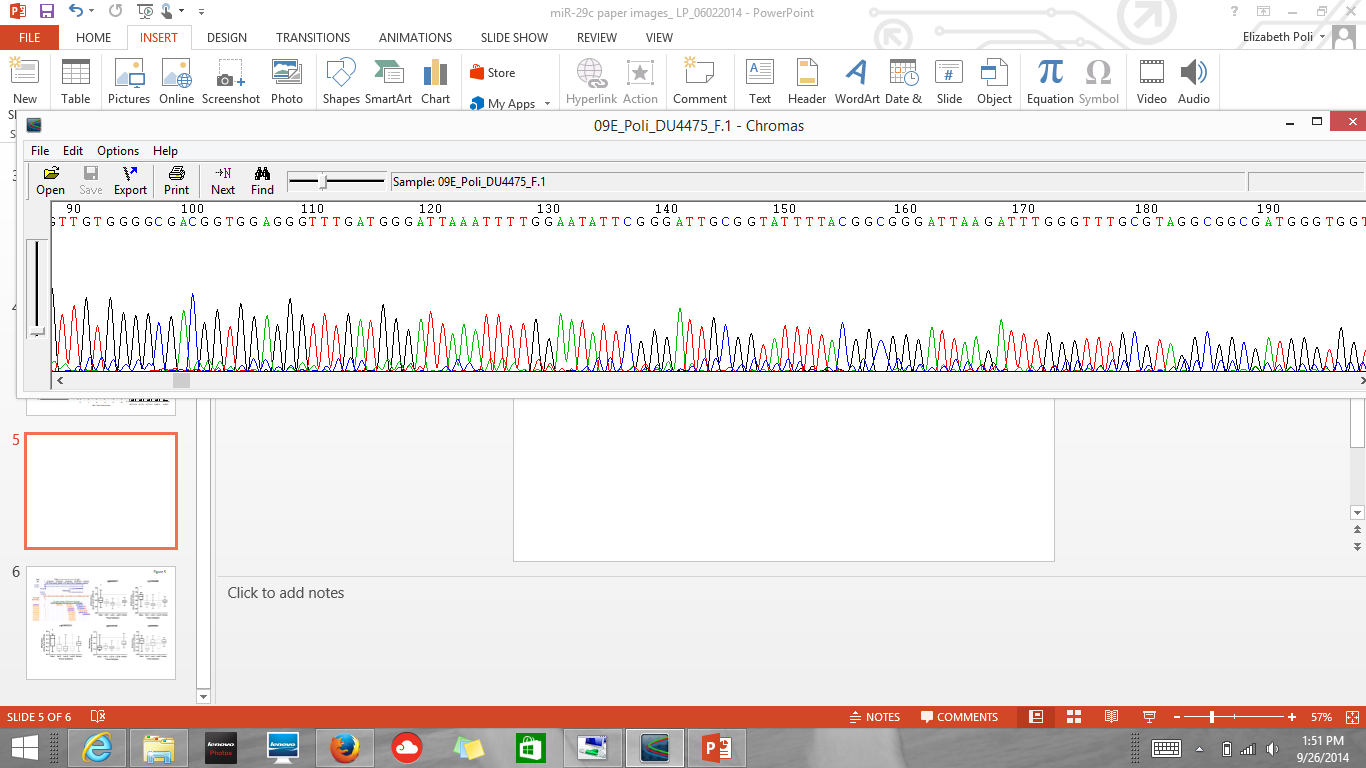


DU4475


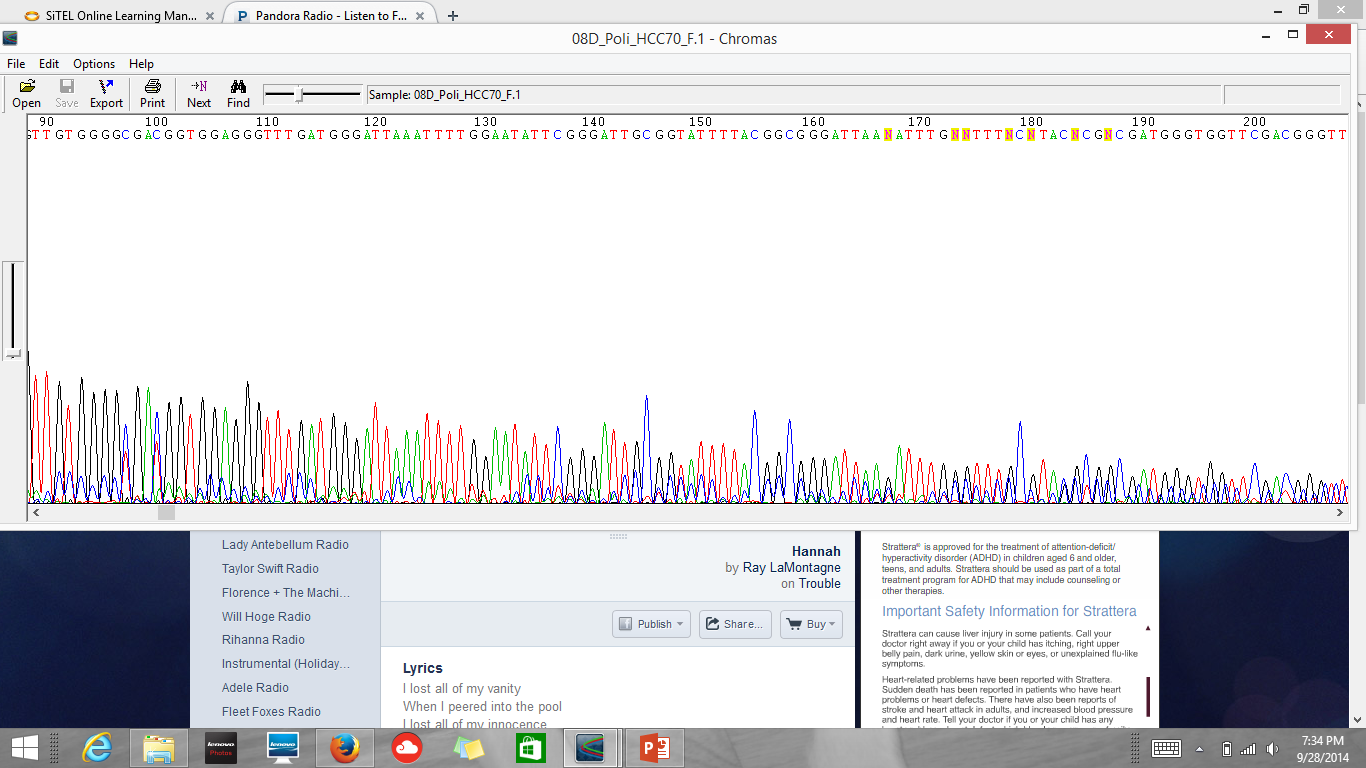


HCC70

HCC1937

HCC202

T47D

ZR7530

Basal-like

Luminal

Supplement: S1 Fig — (DOCX) [file pone.0142224.s001.docx]
