# Supplementary material for: Molecular Subtype-Specific Expression of MicroRNA-29c in Breast Cancer Is Associated with CpG Dinucleotide Methylation of the Promoter
Source: PLoS One. 2015 Nov 5;10(11):e0142224. doi: 10.1371/journal.pone.0142224 (PMC4634951; doi:10.1371/journal.pone.0142224)
Supplement: S1 Table — (DOCX) [file pone.0142224.s002.docx]

**S1 Table. Cell lines and cell culture conditions used**

| Cell Line | Source | Validation through US STR^1^/species/mycoplasma | Media | Cells/ 100mm dish | Cell/ well of 6 well plates | Cells/ T75 flask |
| --- | --- | --- | --- | --- | --- | --- |
| T47D | ATCC HTB-133 | yes/yes/yes | 90% RPMI 10% FBS^2^  1% HEPES 1% P/S^3^ | 1X10^6 | 3X10^5 | 3X10^6 |
| 184A1 | ATCC CRL-8798 | no/no/yes | MEGM BulletKit 0.005 mg/ml transferrin 1ng/ml chloera toxin | 1X10^6 | 3X10^5 | 5X10^6 |
| HCC70 | ATCC CRL-2315 | no/no/yes | 90% RPMI 10% FBS^2^  1% HEPES 1% P/S^3^ | 1X10^6 | 3X10^5 | 5X10^6 |
| DU4475 | ATCC HTB-123 | yes/yes/yes | 90% RPMI 10% FBS^2^  1% HEPES 1% P/S^3^ | 1X10^6 | 3X10^5 | 5X10^6 |
| HCC1428 | ATCC CRL-2327 | yes/yes/yes | 90% RPMI 10% FBS^2^  1% HEPES 1% P/S^3^ | 1X10^6 | 3X10^5 | 5X10^6 |
| AU565 | ATCC CRL-2351 | no/no/yes | 90% RPMI 10% FBS^2^  1% HEPES 1% P/S^3^ | 1X10^6 | 3X10^5 | 5X10^6 |
| ZR7530 | ATCC 1504 | no/no/yes | 90% RPMI 10% FBS^2^  1% HEPES 1% P/S^3^ | 1X10^6 | 3X10^5 | 5X10^6 |
| HCC1937 | ATCC CRL-2336 | yes/yes/yes | 90% RPMI 10% FBS^2^ 1% HEPES 1% P/S^3^ 5%CO2 | 1X10^6 | 5X10^5 | 5X10^6 |
| UACC3199 | University of Arizona Cancer Center | yes/yes/yes | 90% RPMI 10% FBS^2^ 1% HEPES 1% P/S^3^ 5%CO2 | 1X10^6 | 3X10^5 | 5X10^6 |
| HS578T | ATCC HTB-126 | no/no/yes | 90% DMEM  10% FBS^2^  1% P/S^3^ | 1X10^6 | 3X10^5 | 5X10^6 |
| HCC38 | ATCC CRL-2314 | no/no/yes | 90% RPMI 10% FBS^2^ 1% HEPES 1% P/S^3^ | 1X10^6 | 3X10^5 | 5X10^6 |
| MDA-MB-231 | ATCC HTB-26 | no/no/yes | 90% RPMI 10% FBS^2^ 1% HEPES 1% P/S^3^ | 1X10^6 | 3X10^5 | 3X10^6 |
| HMEC | LONZA | Certificate of analysis from Lonza | MEGM BulletKit | 1X10^6 |  | 1X10^6 |

^1^STR: Short tandem repeat, ^2^Fetal Bovine Serum, ^3^Penicillin- Streptomycin
