# Supplementary material for: Molecular Subtype-Specific Expression of MicroRNA-29c in Breast Cancer Is Associated with CpG Dinucleotide Methylation of the Promoter
Source: PLoS One. 2015 Nov 5;10(11):e0142224. doi: 10.1371/journal.pone.0142224 (PMC4634951; doi:10.1371/journal.pone.0142224)
Supplement: S2 Table — (DOCX) [file pone.0142224.s003.docx]

**S2 Table. Bisulfite Sequencing Primers**

| 4132F  WT: GTTCAGAGATCTGTCAGGGGCCAGAGGCCT  BSeq: GTTTAGAGATTTGTTAGGGTTAGAGGTTT  4440R  WT: CCCCTGATCCCAGCAAATACATAC  BSeq: CCCCTAATCCCAACAAATACATAC | 308 bp |
| --- | --- |

WT: wild type , BSeq: bisulfite sequencing
