# Supplementary material for: Molecular Subtype-Specific Expression of MicroRNA-29c in Breast Cancer Is Associated with CpG Dinucleotide Methylation of the Promoter
Source: PLoS One. 2015 Nov 5;10(11):e0142224. doi: 10.1371/journal.pone.0142224 (PMC4634951; doi:10.1371/journal.pone.0142224)
Supplement: S1 Text — (DOCX) [file pone.0142224.s004.docx]

**S1 Text. Analysis of breast cancer cell line FISH**

The normal lymphocyte cell line GM14467 and the normal human mammary epithelial cells (HMEC) each had 2 intact copies of Chromosome 1 with a signal for CEP1 and *miR-29c*. UACC3199 basal-like cell line had five Chromosome 1 derivatives without miR-29c signals. There were two signals for miR-29c translocated on two marker chromosomes. Because of these chromosomal abnormalities, the miR-29c:CEP1 ratio of .4 does not represent deletion. The basal-like cell line HCC1937 had two intact chromosome 1’s, and displayed two extra copies of *miR-29c* on that were duplicated and translocated on two different marker chromosomes. This resulted in an equivocal *miR-29c*:CEP1 ratio of 1.9. HCC70, another basal-like cell line, had four Chromosome 1 copies with intact CEP1 and *miR-29c* signals. This cell line also displayed a CEP1 signal on a marker chromosome, and a dicentric chromosome containing two CEP1 signals and one *miR-29c* signal in 50% of the cells. Twenty-seven percent of the cells did not contain the marker chromosome, and, therefore, contained a balanced polysomy. This cell line has a gain of *mir-29c*, but does not have amplification of the gene. The basal-like cell line DU4475 displayed 4 normal Chromosome 1 copies, 1 marker chromosome with a CEP1 signal but *miR-29c* loss, and 2 marker Chromosome 1 copies that contain two copies of *miR-29c* on each arm of the chromosome *.*  The luminal cell line T47D displays two intact Chromosome 1 copies, and 1 marker chromosome with intact CEP1 and *miR-29c* signals, as well as a *miR-29c* duplication and inversion. These chromosomal abnormalities represent low polysomy, gene gain with a duplication of *miR-29c*, but no amplification. AU565, a luminal cell line, has four intact copies of Chromosome 1, as well as a dicentric chromosome containing two copies of *miR-29c*, which results in a balanced polysomy. The claudin-low cell line HCC38 displayed trisomy of Chromosome 1 and a marker chromosome containing a CEP1 signal. The claudin-low cell line MDAMB231 displays a classic trisomy of Chromosome 1.
